# Self-Regulation of Cerebral Metabolism and Its Neuroprotective Effect After Hypoxic-Ischemic Injury: Evidence From ^1^H-MRS

**DOI:** 10.3389/fnana.2021.672412

**Published:** 2021-06-17

**Authors:** Kexin Li, Yang Zheng, Xiaoming Wang

**Affiliations:** Department of Radiology, Shengjing Hospital of China Medical University, Shenyang, China

**Keywords:** hypoxic-ischemic injury, energy metabolism, amino acid metabolism, neurogenesis, neural plasticity

## Abstract

^1^H-MRS technology can be used to non-invasively detect the content of cerebral metabolites, to assess the severity of hypoxic-ischemic (HI) injury, and to predict the recovery of compromised neurological function. However, changes to the cerebral self-regulation process after HI are still unclear. This study investigated the changes in cerebral metabolites and the potential relationship with the number of neurons and neural stem/progenitor cells (NSPC) using ^1^H-MRS, and finally clarifies the self-regulation of cerebral metabolism and neuroprotection after HI injury. Newborn Yorkshire pigs (28 males, 1.0–1.5 kg) aged 3–5 days were used for the HI model in this study. The pigs were randomly divided into the HI group (*n* = 24) and the control group (*n* = 4), then the experimental group was subdivided according to different recovery time after HI into the following groups: 0–2 h (*n* = 4), 2–6 h (*n* = 4), 6–12 h (*n* = 4), 12–24 h (*n* = 4), 24–48 h (*n* = 4), and 48–72 h (*n* = 4). Following the HI timepoints, ^1^H-MRS scans were performed and processed using LCModel software, and brain tissue was immunohistochemically stained for Nestin and NeuN. Immunofluorescence staining of creatine phosphokinase-BB (CK-BB), N-acetylaspartylglutamate synthetase (NAAGS), glutamate carboxypeptidase II (GCP-II), glutamate-cysteine ligase catalytic subunit (GCLC), glutathione synthase (GS), and excitatory amino acid carrier 1 (EAAC1) was then performed. The ^1^H-MRS results showed that cerebral N-acetylaspartylglutamate (NAAG), glutathione (GSH), and creatine (Cr) content reached their peaks at 12–24 h, which was consistent with the recovery time of hippocampal NSPCs and neurons, indicating a potential neuroprotective effect of NAAG, GSH, and Cr after HI injury.

## Introduction

Hypoxic-ischemic (HI) injury is one of the major causes of neonatal encephalopathy, mainly causes damage to the cerebral cortex, hippocampus, basal ganglia, and thalamus and can lead to complications such as cerebral palsy, epilepsy, and cognitive impairment (Kurinczuk et al., 2010; Wu et al., 2019). The critical step of HI injury is mitochondrial metabolism failure resulting in the rapid consumption of adenosine triphosphate (ATP) and phosphocreatine (PCr) (Johnston et al., 2011; Hagberg et al., 2014; Wisnowski et al., 2016). During HI injury, energy metabolism and the regulation of neurotransmitters affect each other. Compromised intracellular oxidative phosphorylation (OXPHOS), impaired energy metabolism, accumulated glutamic acid (Glu) in the synaptic cleft, overactivated glutamate receptors, overloaded intracellular calcium, and accumulated reactive oxygen species (ROS) after HI all cause damage to neural cells (Pregnolato et al., 2019; Qin et al., 2019).

After nerve injury, the brain's function and structure can undergo adaptive changes, including regulation at the molecular, cellular, and physiological aspects. At the developmental stage, neonatal brain and its adaptation to injury is stronger than that of adult brains (Rocha-Ferreira and Hristova, 2016). During the cerebral self-regulation process, metabolites are the functional products for gene expression, and they also act as the neurotransmitters that regulate cell signal transduction (Blaise et al., 2017). When ATP is insufficient, PCr can transfer its high-energy phosphate to adenosine diphosphate (ADP) under the catalysis of creatine kinase (CK), further generating creatine (Cr) and ATP to provide energy and thereby playing a vital role in cellular energy homeostasis (Sahlin and Harris, 2011; Wisnowski et al., 2016; Gaddi et al., 2017). The cerebral metabolism of amino acids can also be adjusted accordingly in response to increased Glu and overactivated receptors. For example, N-acetylaspartylglutamate (NAAG) reduces the excitotoxic effect of Glu by competitively binding to type 3 metabotropic glutamate receptors (mGluR3) thus partly reducing cell damage. Additionally, as the important antioxidant in the body, glutathione (GSH) can protect cells from oxidative damage by removing ROS in a reaction catalyzed by glutathione peroxidase (Thorwald et al., 2019). Another process, mediated by the malate-aspartate shuttle (MAS), not only includes amino acid conversion but is also closely related to energy metabolism. After shuttling into the mitochondria, malic acid and Glu are converted into aspartic acid (Asp) and α-ketoglutarate, which is tightly coupled with the NAD^+^/NADH electron transport chain in neurons and further provides cellular energy (Xu et al., 2020).

^1^H-MRS imaging combined with LCModel software can be used to non-invasively detect the changes in cerebral metabolite content and quantitatively analyze the concentrations of these metabolites (Moss et al., 2018; Dhamala et al., 2019). Through the continuous optimization of scanning and post-processing techniques, metabolites with similar molecular structures and spectral characteristics (N-acetylaspartate (NAA)/NAAG, Glu/glutamine (Gln), etc.) can also be quantified separately (Menshchikov et al., 2020). This study aims to investigate the changes in cerebral metabolites and their potential relationship with the number of neurons and NSPCs by ^1^H-MRS, and reveals how self-regulation of cerebral metabolism contributes to neuroprotection after HI injury.

## Materials and Methods

### Experimental Animals

Twenty-eight newborn Yorkshire pigs (male, 1.0–1.5 kg) aged 3–5 days were selected and randomly divided into the experimental group (*n* = 24) and the control group (*n* = 4). According to the recovery time after HI, the experimental group was divided into 6 subgroups, including 0–2 h (*n* = 4), 2–6 h (*n* = 4), 6–12 h (*n* = 4), 12–24 h (*n* = 4), 24–48 h (*n* = 4), and 48–72 h groups (*n* = 4). The relevant procedures for experimental animals were performed in accordance with the *Laboratory Animal Care and Use Guidelines* issued by the National Research Council and approved by the Animal Care and Use Institutional Committee (approval number 2015PS337K).

### Animal Modeling

#### Anesthesia and Mechanical Ventilation

Anesthesia was administered using Sumianxin (Changchun Institute of Military Medical Research, Changchun, China) injected intramuscularly at a dose of 0.6 ml/kg. After the animal's corneal reflex disappeared, tracheal intubation (diameter 2.5 mm) and ventilator-assisted breathing (U-25T bi-level airway pressure ventilator; Tianjin Yihejiaye Medical Technology Co., Ltd., China) was conducted with the respiratory rate set at 10 times/min and the pressure at 15–25 hPa. A Heal Force pulse oximeter (Shenzhen Hexin Zhondian Medical Equipment Co., Ltd., China) was used to monitor vital sign (pulse and blood oxygen saturation). An indwelling trocar in the right ear vein served as the venous channel.

#### Common Carotid Artery Dissection

Skin was disinfected three times with iodophor followed by a median cervical incision, blunt dissection was performed in layers, and the common carotid artery was dissociated, with special attention paid to avoid damage to the carotid sinus and vagus nerve during the operation.

HI injury: The bilateral common carotid arteries were located by surgical sutures and occluded by artery clamps. Pigs mechanically inhaled a mixture of 6% oxygen and 94% nitrogen (Dalian Special Gas, Dalian, China) at the same time. After 40 min, the artery clamps were removed, 100% oxygen was resumed, and the cervical incision was sutured. The ventilator was then stopped and the tracheal tube was removed after spontaneous breathing resumed (Zheng and Wang, 2017).

The animals in the control group underwent preoperative anesthesia and common carotid artery dissociation, while those in the experimental group received all surgical procedures including HI. The room temperature was kept at 28–30°C during the operation.

### ^1^H-MRS Scan and Data Post-processing

^1^H-MRS scans were performed after the surgical procedures were completed for the experimental group and the control group. An Achieva 3.0T magnetic resonance scanner (Philips Healthcare, the Netherlands) was employed as the MR equipment has gradient coil transmission and 8-channel head coil reception. A single voxel sequence was utilized for ^1^H-MRS, and the scanning parameters were as follows: TR = 2,000 ms; *TE* = 37 ms; *NSA* = 64; *VOI* = 10 × 10 × 10 mm. The region of interest was selected in the right basal ganglia. The LCModel software package was used to analyze the ^1^H-MRS results (Zheng and Wang, 2018) (Cr+PCr at 3.02 ppm, NAA+NAAG at 2.02 ppm, Glu at 2.04–2.35 ppm and 3.75 ppm, Gln at about 2.35 ppm, Asp at 2.71 ppm, and GSH at 2.95 ppm [Moss et al., 2018)].

### Immunohistochemistry and Immunofluorescence Staining

The brain tissue was fixed with formalin, embedded in paraffin and sliced into 4 mm-thick coronal sections containing hippocampus, basal ganglia, and cortex. Immunohistochemistry staining was performed automatically using a BOND-MAX^TM^ automatic staining machine (Leica, Germany) (Li et al., 2018). The primary antibodies used were NeuN (Abcam, ab128886) and Nestin (Abcam, ab92391). Three high-magnification fields of view (×400) were randomly selected from each encephalic region, and the protein expression and the number of protein-positive cells were measured with ImageJ software (Java1.6.0, National Institutes of Health).

Immunofluorescence staining was performed to detect creatine phosphokinase-BB (CK-BB), NAAG synthetase (NAAGS), glutamate carboxypeptidase II, (GCP-II), glutathione synthase (GS), glutamate-cysteine ligase catalytic subunit (GCLC), and excitatory amino acid carrier-1 (EAAC1). The tissue sections were deparaffinized with xylene and hydrated with gradient ethanol, followed by antigen retrieval with microwave heating for 37 min using citrate buffer (0.01 M, pH 6.0). The sections were then incubated with normal goat serum at room temperature for 40 min to block non-specific antibody binding, and followed by incubation of the sections overnight at 4°C with the following selected primary antibodies: rabbit polyclonal antibody to RIMKA (diluted concentration 1:100, abs134743); rabbit polyclonal antibody to PSMA (diluted concentration 1:50, ab58779); rabbit polyclonal antibody to Creatine kinase B type (diluted concentration 1:100, ab151579); rabbit polyclonal antibody to GCLC (diluted concentration 1:100, ab53179); rabbit monoclonal antibody to GS, (diluted concentration 1:100, ab124811), and rabbit polyclonal antibody to EAAT3 (diluted concentration 1:50, 12686-1-AP). Goat anti-rabbit IgG labeled with Alexa Fluor 488 (diluted concentration 1:100, ImmunoWay, RS3211) was used as the secondary antibody and incubated with the sections at room temperature for 4 h. Finally, nuclear staining was performed by incubation with 4′,6-Diamidino-2-Phenylindole, Dihydrochloride (DAPI) for 5 min. The immunofluorescence images were collected using a confocal laser scanning microscope (LSM880; ZEISS, Gottingen, Germany) (×400) and the mean fluorescence intensity was measured using ImageJ software (Java1.6.0; National Institutes of Health).

### Statistical Analysis

A one-way ANOVA analysis of variance and the LSD test were employed to compare the differences between the subgroups with *P* < 0.05 considered to be statistically significant. Pearson's correlation analysis was used to analyze the relationship among metabolites, and all statistical analyses were conducted using SPSS (version 22.0; IBM, New York) and GraphPad Prism (version 8.0.2; GraphPad Software, California). The correlation heatmap was drawn with Origin Pro software (9.7.0.188; OriginLab Corporation, Northampton, MA, USA).

## Results

### Changes Observed in Cerebral Metabolites After HI Injury by ^1^H-MRS

**Figure 1:**
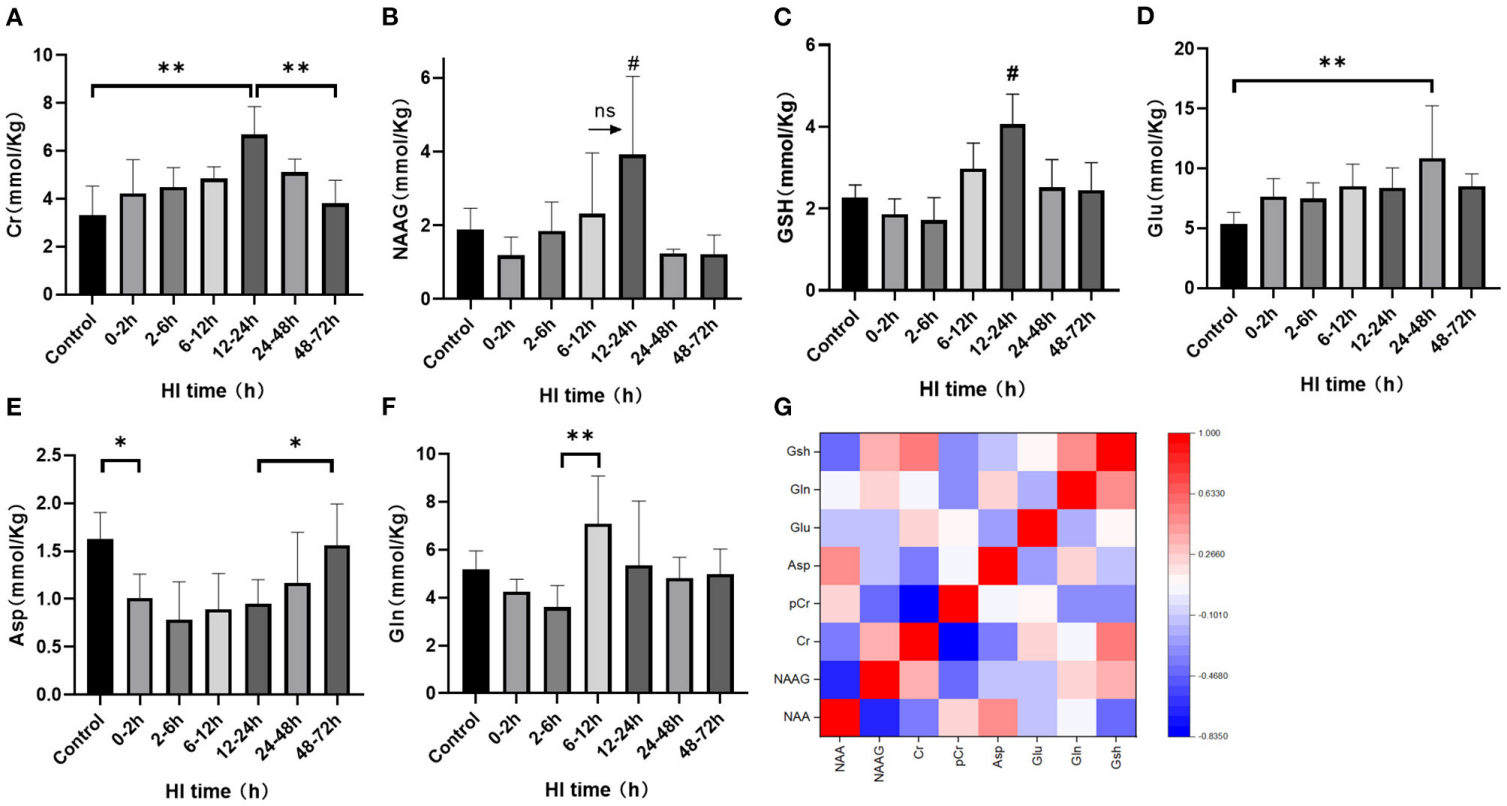
The changes in cerebral metabolites discovered using ^1^H-MRS and the correlation heatmap after HI injury. **(A–F)** Changes in Cr, NAAG, GSH, Glu, Asp, and Gln after HI injury (**P < 0.01, *P < 0.05, ns *P* > 0.05,# compared with other groups, *P* < 0.05). Data are expressed as mean ± standard deviation. **(G)**. Correlation heatmap of cerebral metabolites after HI injury. Red indicates a positive correlation and blue a negative correlation with darker colors suggesting a stronger correlation.

^1^H-MRS was used to observe the cerebral amino acid metabolism changes at different time periods after HI injury, as shown in Figures 1A–F. There was no statistical difference for the cerebral NAAG content between the 6–12 h and 12–24 h groups (*P* = 0.057; LSD test), but they both were significantly higher than those of the other groups (*P* < 0.05; LSD test). Asp content was significantly reduced at 0–2 h (*P* = 0.032; LSD test), and increased at 48–72 h compared with the 12–24 h group (*P* = 0.047; LSD test). Compared with the others, GSH content increased significantly in the 12–24 h group (*P* < 0.05; LSD test). Cr content in the 12–24 h group was significantly higher than that in the control group (*P* < 0.001; LSD test) and the 48–72 h group (*P* = 0.001; LSD test). Glu content in the 24–48 h group was significantly higher than that in the control group (*P* = 0.002; LSD test). Gln content in the 6–12 h group was significantly higher than that in the 2–6 h group (*P* = 0.003; LSD test). The correlation heatmap for each metabolite is shown in Figure 1G. After HI injury, the NAAG and NAA content were significantly negatively correlated (*r* = −0.661, *P* < 0.001) as was Cr and PCr (*r* = −0.833, *P* < 0.001). In contrast, Gln and GSH were significantly positively correlated (*r* = 0.462, *P* = 0.020) and there was a significant positive correlation between Cr and GSH (*r* = 0.521, *P* = 0.011).

### Altered Expression of CK-BB, NAAGS, and GCP-II After HI Injury

**Figure 2:**
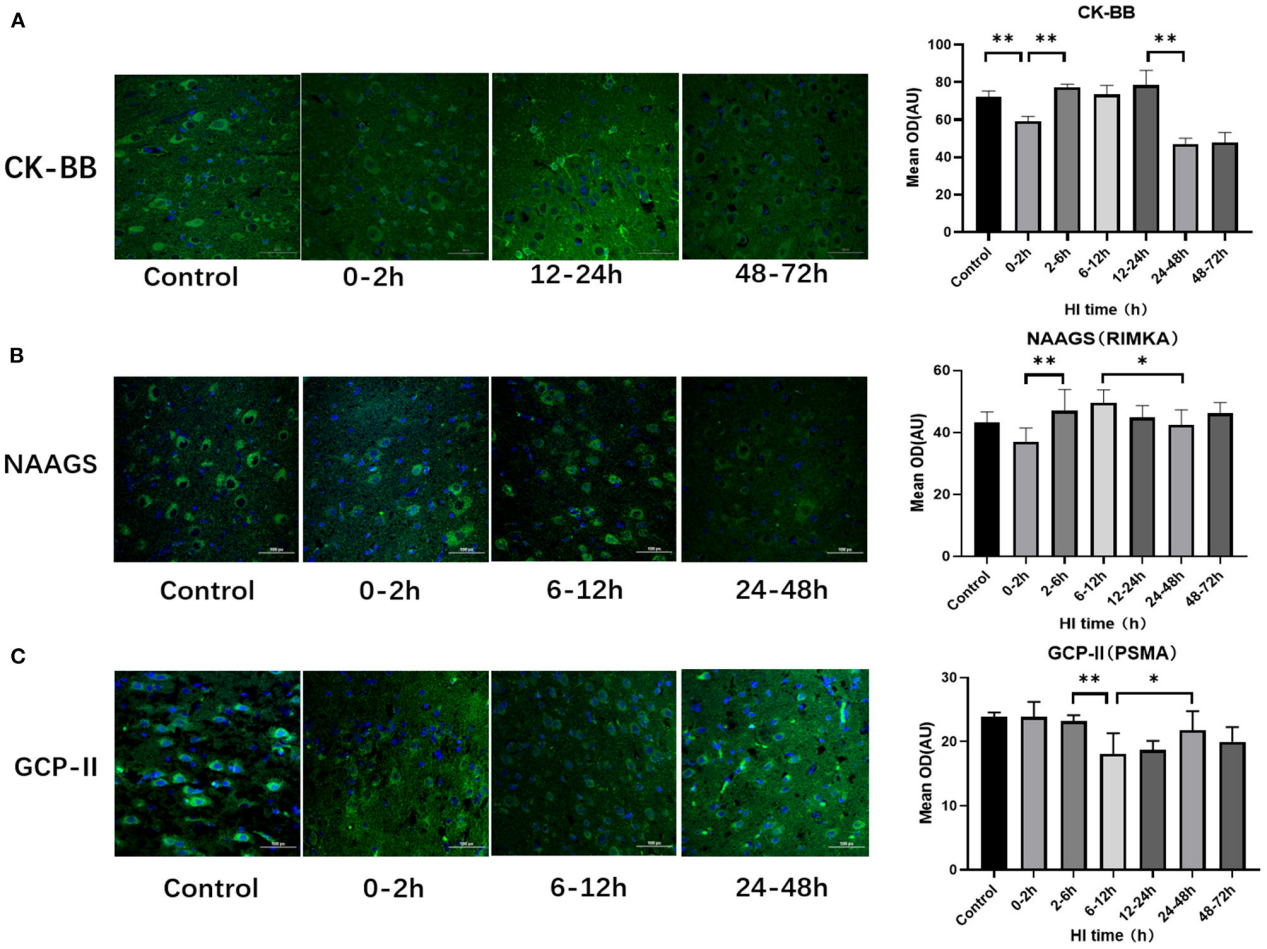
Differences in the expression of CK-BB, NAAGS, and GCP-II after HI injury (×400). **(A)** Tendency chart for cerebral CK-BB staining and mean integrated optical density in the control group and the experimental groups at 0–2 h, 12–24 h, and 48–72 h after HI injury. Green fluorescence represents CK-BB. Compared with the control group, the fluorescence intensity decreases at 0–2 h, increases at 2-6 h, 6-12 h, 12-24 h and then decreases again at 48–72 h after HI injury. **(B)** Tendency chart for cerebral NAAGS staining and mean integrated optical density in the control group and the experimental groups at 0–2 h, 6–12 h, and 24–48 h after HI injury. Green fluorescence represents NAAGS. The fluorescence intensity of NAAGS reaches its peaks at 2–6 h and 6–12 h after HI injury. **(C)** Tendency chart for cerebral GCP-II staining and mean integrated optical density in the control group and the experimental groups at 0–2 h, 6–12 h, and 24–48 h after HI injury. Green fluorescence represents GCP-II. The fluorescence intensity of GCP-II decreases at 6–12 h, increases at 24–48 h after HI injury. **P < 0.01, *P < 0.05, and data are expressed as mean ± standard deviation.

There were differences of CK-BB, NAAGS, GCP-II expression among subgroups after HI injury (*P* < 0.001, *P* = 0.027, and *P* = 0.002, respectively; ANOVA). The differences in CK-BB, NAAGS, and GCP-II expression after HI injury are shown in Figure 2. The expression of CK-BB decreased significantly at 0–2 h (*P* < 0.001; LSD test), increased at 2–6 h (*P* < 0.001; LSD test), and continued to increase at 12–24 h after HI injury. Compared with the 0–2 h group, the expression of NAAGS increased significantly at 2–6 h, 6–12 h and 12–24 h (*P* = 0.006, *P* = 0.001, and *P* = 0.024, respectively; LSD test). The expression of NAAGS in the 24–48 h group was significantly lower than that in the 6–12 h group (*P* = 0.041; LSD test). Compared with the 2–6 h group, the expression of GCP-II decreased significantly at 6–12 h (*P* = 0.003; LSD test), and then increased at 24–48 h (*P* = 0.025; LSD test)

### Altered Expression of EAAC1, GCLC, and GS After HI Injury

**Figure 3:**
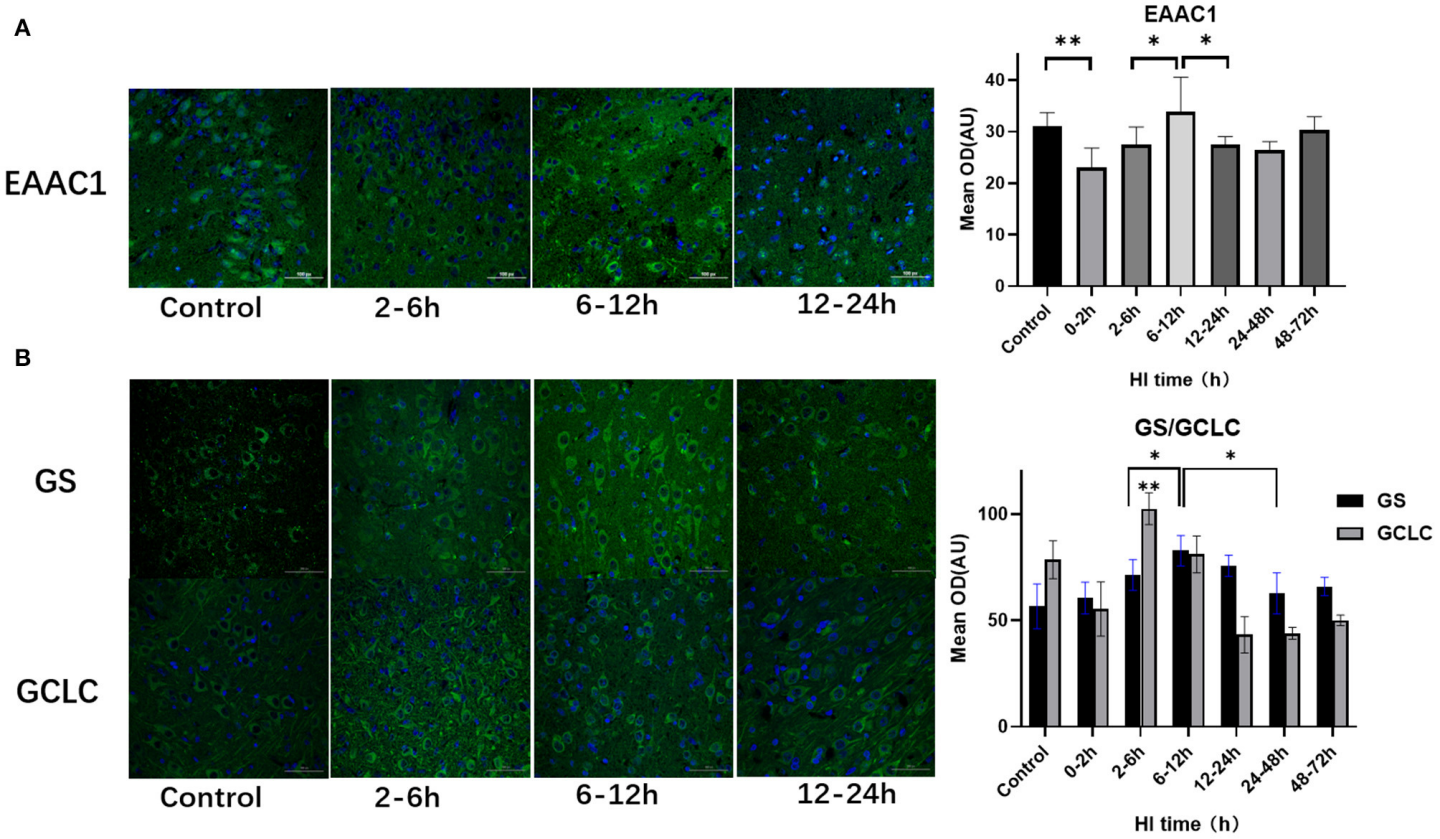
Differences in the expression of EAAC1, GS, and GCLC after HI injury (×400). **(A)** Tendency chart for cerebral EAAC1 staining and mean integrated optical density in the control group and the experimental groups at 2–6 h, 6–12 h, and 12–24 h after HI injury. Green fluorescence represents EAAC1. Compared with the control group, the fluorescence intensity in the experimental group decreases at 0–2 h, increases at 6-12 h, and then decreases again at 12–24 h after HI injury. **(B)** Tendency chart for cerebral GCLC and GS staining and mean integrated optical density in the control group and the experimental groups at 2–6 h, 6–12 h, and 12–24 h after HI injury. Green fluorescence represents GCLC and GS. The fluorescence intensity of GCLC and GS reach their peaks at 2–6 h and 6–12 h after HI injury, respectively. **P < 0.01, *P < 0.05, and data are expressed as mean ± standard deviation.

EAAC1, GCLC, and GS expression also differed among subgroups after HI injury (*P* = 0.008, *P* < 0.001, *P* = 0.001, respectively; ANOVA). Figure 3 shows the differences in the expression of EAAC1, GCLC and GS after HI injury. The expression of EAAC1 decreased significantly at 0–2 h (*P* = 0.004; LSD test), increased at 6–12 h compared with the 2–6 h group (P = 0.018; LSD test), and decreased at 12–24 h after HI injury (*P* = 0.019; LSD test). GS expression tended to increase first and then decrease with a peak at 6–12 h (*P* < 0.05; LSD test), a slight decrease at 12–24 h, with no statistical difference (*P* = 0.203; LSD test), and significantly decreased at 24–48 h after HI injury (*P* = 0.026; LSD test). The peak expression of GCLC was at 2–6 h after HI, and it was significantly higher than that of the other groups (*P* < 0.01; LSD test).

### Hippocampal NSPC Numbers Varied After HI Injury

**Figure 4:**
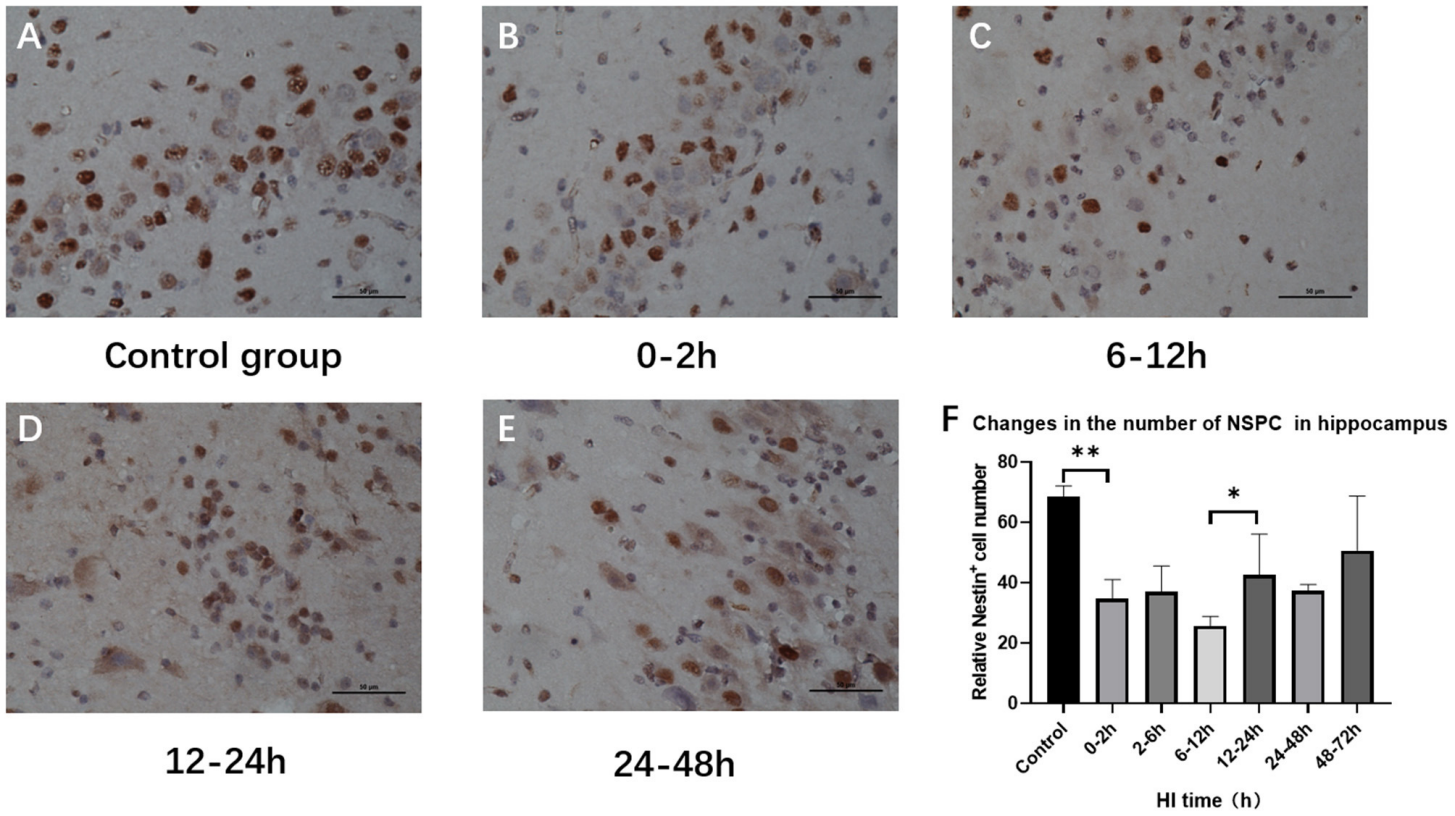
Variations in the number of hippocampal NSPCs after HI injury (×400). **(A)** Nestin immunohistochemical staining in the control group. **(B–E)** Nestin immunohistochemical staining at 0–2 h, 6–12 h, 12–24 h, and 24–48 h after HI. The number of Nestin-positive cells in the hippocampus decreases first and then increases. **(F)** The number of Nestin-positive cells decreases at 0–2 h after HI injury compared with the control group, and it significantly increases in the 12–24 h group compared with the 6–12 h group. **P < 0.01, *P < 0.05, and data are expressed as mean ± standard deviation.

Hippocampal NSPC numbers varied among subgroups after HI injury (*P* = 0.002; ANOVA). Compared with the control group, the number of hippocampal NSPCs decreased significantly in the 0–2 h, 2–6 h, and 6–12 h groups (*P* = 0.001, *P* = 0.001, *P* < 0.001, respectively; LSD test), with a rebound in the 12–24 h group (*P* = 0.049; LSD test) and there was no statistical difference compared with the 24–48 h and 48–72 h group (*P* = 0.113; LSD test) (Figure 4).

### Changes in the Number of Hippocampus and Basal Ganglia Neurons After HI Injury

**Figure 5:**
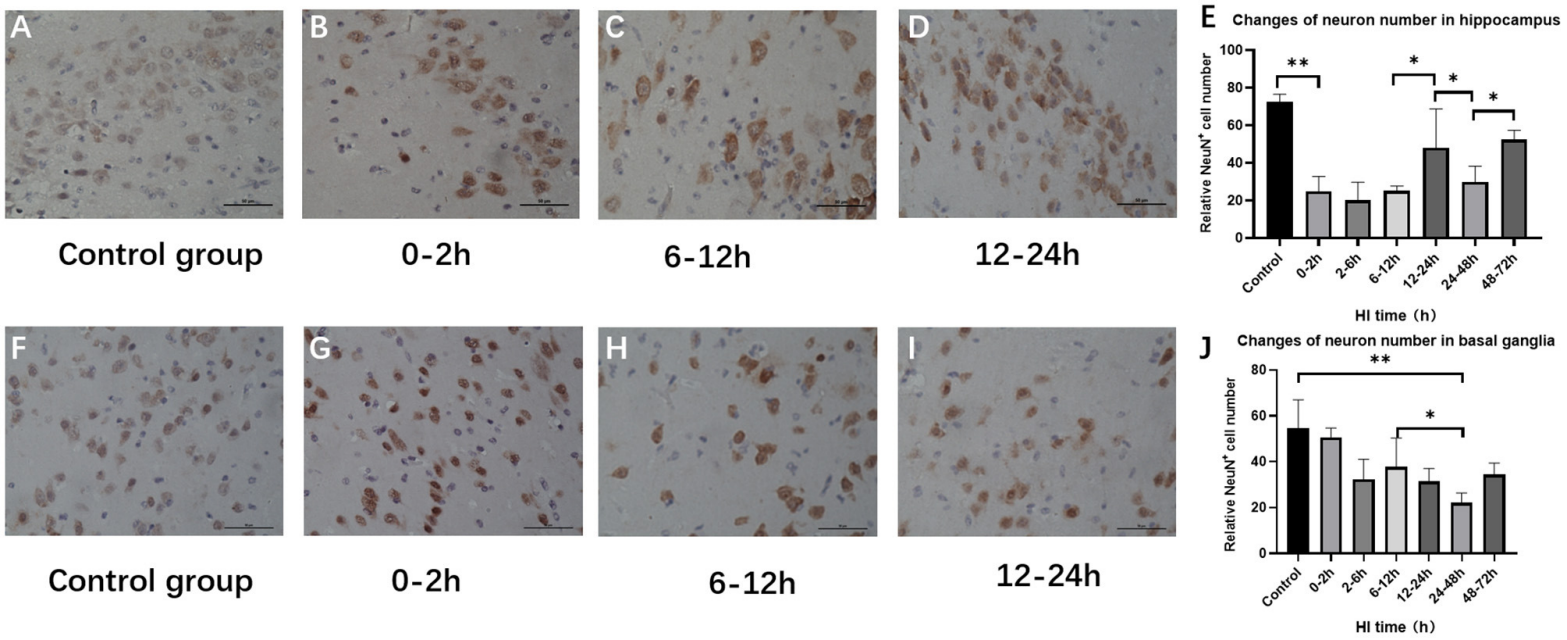
Changes in the number of NeuN-positive cells in the hippocampus and basal ganglia after HI (×400). **(A)** Immunohistochemical staining of hippocampal NeuN in the control group. **(B–D)** Immunohistochemical staining of hippocampal NeuN at 0–2 h, 6–12 h, and 12–24 h after HI injury. **(E)** The number of NeuN-positive cells in the hippocampus decreases at 0–12 h and increases significantly at 12–24 h after HI injury. **(F)** NeuN immunohistochemical staining of the basal ganglia in the control group. **(G–I)** NeuN immunohistochemical staining of the basal ganglia at 0–2 h, 6–12 h, and 12–24 h after HI injury. **(J)** Compared with the control group, there is no significant difference in the number of NeuN-positive cells in the basal ganglia at 0–6 h after HI injury, and the number of NeuN-positive cells at 24–48 h after HI injury is significantly reduced. **P < 0.01, *P < 0.05, and data are expressed as mean ± standard deviation.

The numbers of hippocampus and basal ganglia neurons were significantly changed among control groups and HI groups (*P* < 0.001, *P* = 0.004; ANOVA). Hippocampal neurons were significantly reduced at 0–12 h (*P* < 0.01; LSD test), but increased at 12–24 h after HI (*P* = 0.014; LSD test), and reduced at 24–48 h after HI (*P* = 0.042, LSD test). Compared with the control group, the number of basal ganglia neurons was significantly reduced at 2–6 h (*P* = 0.005, LSD test) and was further reduced at 24–48 h after HI (*P* < 0.001; LSD test) as shown in Figure 5.

## Discussion

As a non-invasive method, ^1^H-MRS detects metabolites by measuring their relative quantification (ratio), which will help assess the severity of HI injury and predict the recovery of neurological function. However, the metabolite ratio cannot reflect the specific changes in a single case or the potential mechanisms of energy failure and metabolic disorders after HI injury (McKenna et al., 2015; Barta et al., 2018; Montaldo et al., 2019). Shibasaki et al. (2018) further used ^1^H-MRS imaging to quantitatively assess the severity of brain damage after HI injury and predict the adverse outcomes for the nervous system by proposing corresponding biomarkers and specific cutoff values. Previous studies mainly focused on the anatomical and functional outcomes after HI injury using markers extracted from MRS, but there are only a few focused on the dynamic changes of cerebral metabolism (Locci et al., 2020). In order to study the self-regulation of cerebral metabolism after HI injury and find biomarkers of neuroprotective effects, ^1^H-MRS imaging was used in this study to observe the dynamics of cerebral metabolites in the early stage of HI injury and further examine how this relates to the evolution of the number of neurons in the corresponding period.

### The Relationship of NAAG and GSH and Their Metabolic Processes With the Altered Number of Neurons During HI Injury

The synthesis of NAAG by NAA and Glu is catalyzed by NAAGS. After it is released from vesicles, NAAG activates mGluR3 in the presynaptic membrane and inhibits the release of Glu, thereby exerting neuroprotective effects (Baslow, 2010; Nordengen et al., 2020). The ^1^H-MRS results in the current study showed that NAAG reached a peak at 12–24 h after HI injury and that there was a significant negative correlation with NAA. Histopathological findings indicated that NAAGS expression trended upward at 2–6 h and lasted until 12–24 h after HI injury, suggesting increased NAAG synthesis during this period. There was also an increasing trend for the number of hippocampal neurons at 12–24 h after HI injury, which was consistent with the changes in NAAG and indicating some recovery of hippocampal neurons under the action of it. The number of basal ganglia neurons did not decrease further, which also reflects the neuroprotective effect. However, the action of NAAG is short-lived as it is inactivated immediately by GCP-II after binding to the receptor, which regenerates NAA and Glu and results in a further increase of Glu (Thomas et al., 2010; Neale and Yamamoto, 2020). We also found that NAAGS expression was significantly reduced at 24–48 h and, in the meantime, GCP-II expression was significantly increased, and NAAG content was considerably decreased while Glu content was significantly increased after HI. Furthermore, the number of hippocampal and basal ganglia neurons both decreased, which was due to the decreased production and increased decomposition of NAAG as well as neuronal damage caused by Glu excitotoxicity.

GSH synthesis by neurons requires 3 amino acids (Glu, cysteine, and glycine) and 2 ATP-dependent enzymatic steps (Aoyama et al., 2008). Initially, the transport of cysteine, an important substrate for the synthesis of GSH, is mediated by EAAC1 (Lee et al., 2020); then GCL catalyzes the conjugation of cysteine and Glu to form γ-glutamylcysteine (γ-GC), and finally GS catalyzes the combination of γ-GC with glycine to synthesize GSH (Yu et al., 1999; Lian et al., 2018). Using histopathology, we found that the expression of EAAC1 increased significantly at 6–12 h while GCLC and GS reached their peaks at 2–6 h and 6–12 h after HI injury, respectively, which corresponds with the process of GSH synthesis. ^1^H-MRS results showed that the GSH content reached its peak at 12–24 h after HI injury, indicating that EAAC1, GCLC, and GS were involved in GSH synthesis in the brain after HI injury. Increased GSH can reduce ROS levels in a stress state, thus reducing neuronal damage (Song et al., 2014). This study found that after HI injury, the time of peak GSH was consistent with that of the increased number of hippocampal neurons, indicating that GSH can reflect neuron recovery to some extent. However, more details on the protective effect of GSH need to be confirmed by subsequent experiments.

### Effect of Cr and Its Metabolic Process on the Number of Hippocampal NSPCs and GSH Synthesis During HI Injury

In the past, cerebral Cr content was considered to be relatively constant, thus it is often used as an internal reference for the relative quantification of metabolites. In recent years, studies have found that Cr dynamically changes, and its content is related to physiological activity or vascularization in a specific area (Rae, 2014; McKenna et al., 2015). In this study, ^1^H-MRS was employed to observe changes in Cr content after HI injury. The results showed a gradual increase at first, which peaked at 12–24 h, and then a decrease; this variation depends on the conversion from PCr and the catalytic enzyme activity of CK-BB. The ^1^H-MRS results in this study showed a significant negative correlation between Cr and PCr, and histopathology found that CK-BB expression increased at 2–6 h and lasted 12–24 h after HI injury. These results indicate that there is a mutual conversion between PCr and Cr during this period.

Studies have confirmed that the PCr-Cr system can compensate for the decrease in ATP caused by hypoxia or ischemia to a certain extent (Gaddi et al., 2017), and the increased Cr content has a neuroprotective effect under hypoxic conditions (Rae, 2014). There is some recovery of mitochondrial function at 6–24 h after HI injury (Yin et al., 2008; Rocha-Ferreira and Hristova, 2016; Thornton et al., 2018) and, in the meantime, the increased Cr can improve the efficiency of OXPHOS in mitochondria (Lowe et al., 2015). This then provides ATP for the proliferation and differentiation of NSPCs as well as neuron regeneration (Arrazola et al., 2019; Torres-Cuevas et al., 2019). This study further analyzed the relationship between Cr and hippocampal NSPC variation after HI injury by histopathology. The number of NSPCs in the hippocampus showed a tendency to decrease first and then increase at 12–24 h after HI injury. At the initial 0–12 h timepoint, the number of NSPCs in the hippocampus decreased, then significantly increased at 12–24 h after HI injury. This was consistent with the time of the Cr increase, indicating which can provide information about the recovery of energy metabolism and the change in number of NSPCs after HI injury.

In addition, this study found a significant positive correlation between the Cr content and GSH, suggesting that a correlation between energy metabolites and amino acid metabolites also exists. Since, GSH synthesis is dependent on ATP (Aoyama et al., 2008), the PCr-Cr shuttle system may provide ATP for it after HI injury. The results showed that the 2 metabolites were moderately related, which may be because creatine metabolism is not the only way to provide energy after HI injury. For example, the pentose phosphate pathway (PPP) is also increased during oxidative stress and is necessary for the regeneration of GSH (Amaral et al., 2010; Brekke et al., 2015). However, the detailed influence and action of energy metabolism on amino acid metabolism needs to be further studied.

This study also found an increased number of neurons in the hippocampus at 48–72 h after HI injury. Meanwhile, the Glu content decreased while the Asp content increased significantly, which may be due to the effect of MAS. The MAS shuttling of Glu into the mitochondria and the increase of Asp and α-ketoglutarate conversion provide energy for cell regeneration (Beckervordersandforth, 2017), but the specific mechanisms still need to be further confirmed by experimentation.

## Conclusion

Cerebral metabolites including NAAG, GSH, and Cr displayed a transient elevation following HI injury. This trend is consistent with the duration of increased hippocampal neurons and NSPCs, indicating that NAAG, GSH, and Cr may self-regulate and have neuroprotective effects after HI injury.

## Data Availability Statement

The raw data supporting the conclusions of this article will be made available by the authors, without undue reservation.

## Ethics Statement

The animal study was reviewed and approved by Animal Care and Use Institutional Committee of Shengjing Hospital.

## Author Contributions

KL: investigation, data curation, and writing-original draft. YZ: validation, resources, and supervision. XW: conceptualization, methodology, writing-review and editing, and project administration. All author participated sufficiently to take public responsibility for its content, read, and approved the submitted version.

## Conflict of Interest

The authors declare that the research was conducted in the absence of any commercial or financial relationships that could be construed as a potential conflict of interest.

## Abbreviations

HI: hypoxic ischemia
ATP: adenosine triphosphate
PCr: phosphocreatine
OXPHOS: oxidative phosphorylation
ADP: adenosine diphosphate
CK: creatine kinase
Cr: creatine
ROS: reactive oxygen species
Glu: glutamic acid
Gln: glutamine
NAAG: N-acetylaspartylglutamate
NAA: N-acetylaspartate
GCP-II: glutamate carboxypeptidase II
EAAC1: excitatory amino acid carrier 1
mGluR3: metabotropic glutamate receptor type 3
GSH: glutathione
MAS: malate-aspartate shuttle
Asp: aspartic acids
CK-BB: creatine phosphokinase-BB
NAAGS: NAAG synthetase
GS: glutathione synthase
GCLC: glutamate-cysteine ligase catalytic subunit
γ-GC: γ-glutamylcysteine
PPP: pentose phosphate pathway.
